# COVID-19 vaccine hesitancy among parents in Low- and Middle-Income Countries: A meta-analysis

**DOI:** 10.3389/fpubh.2023.1078009

**Published:** 2023-02-15

**Authors:** Wafa Abu El Kheir-Mataria, Basma M. Saleh, Hassan El-Fawal, Sungsoo Chun

**Affiliations:** Institute of Global Health and Human Ecology, The American University in Cairo, New Cairo, Egypt

**Keywords:** vaccine hesitancy, COVID-19, Low-Income Countries, Middle-Income Countries, parental

## Abstract

**Background:**

Vaccination is the most effective method to prevent the spread of infectious diseases. Nevertheless, vaccine hesitancy has been an issue. Parental hesitancy toward vaccines is a major part of the problem. COVID-19 vaccine acceptance is no different, it poses another challenge in facing the pandemic. In Low- and Middle-Income Countries (L&MICs) several studies measured parents' acceptance to vaccinate their children against COVID-19 and resulted in different acceptance proportions.

**Aims:**

The paper aims at obtaining a precise estimate of the overall proportion of L&MICs' parents accepting to vaccinate their children against COVID-19 and identifying the main determinant of their decisions.

**Methods:**

This meta-analysis follows the PRISMA 2020 statement on updated guidelines and the checklist for reporting systematic reviews. Studies published between December till February 2022 were assessed for inclusion. The final effect size (i.e., the proportion of parents in L&MICs accepting to vaccinate their children against COVID-19) was measured using the Arcsine proportions method. Analysis was done using R program.

**Results:**

The proportion of parents in L&MICs accepting to vaccinate their children against COVID-19 is 49%. The major reason for their acceptance is their belief that COVID-19 vaccine is fundamental to the fight against the pandemic while the most common factor for parents' hesitancy to vaccinate their children against COVID-19 is their concerns about vaccine efficacy, safety, and possible side effects.

**Conclusion:**

The proportion of parents in L&MICs accepting to vaccinate their children against COVID-19 is lower than the global level. To increase parental acceptance, responsible authorities should concentrate on increasing their population's trust in the government and in vaccine manufacturers. As well as concentrating on increasing acceptance of the vaccine idea in general.

## Introduction

Vaccination is one of the most important achievements in medical and public health history. It has proved to be the most effective method to prevent the spread of infectious diseases (1). Nevertheless, vaccine hesitancy has been an issue for a while now. Some people are reluctant to be vaccinated, which increases their risk for diseases and increases the risk of public threat through diminishing the ability to achieve and sustain “herd immunity” (2). Vaccine hesitancy is defined as the “delay in acceptance or refusal of vaccination despite availability of vaccination services” (3). People's acceptance of a vaccine is known to be influenced by several factors related to the people themselves (e.g., education level, complacency, convenience), to the vaccine (e.g., safety and efficacy) and to external factors (e.g., policies, media, confidence) (4, 5).

People opposing vaccines are called “anti-vaxxers.” Studies have found that anti-vaxxers are mostly mothers who are older in age with higher education and socioeconomic status (6). Since children are normally the largest vaccine recipient group, parental hesitancy toward vaccines is a major part of the problem of stopping the spread of infectious diseases.

COVID-19 is no different case from other infectious diseases when it comes to vaccines. Although COVID-19 has resulted in millions of deaths and the count is still going on. COVID-19 vaccine acceptance proves to pose another challenge in facing the pandemic (7). COVID-19 vaccine acceptance is a worldwide issue (8). However, L&MICs seem to have a special context where certain L&MICs have a higher acceptance rate than some High-Income Countries (HICs) (9). A number of studies address COVID-19 vaccines acceptance in L&MICs (9–11). These studies concluded different acceptance proportions and different factors influencing vaccine acceptance decisions. As for the parents' population, many studies were performed to study parents' acceptance of COVID-19 vaccines and the determinants of their behavior. However, many of these studies were conducted in HICs (12–14).

Given the difference in the COVID-19 vaccine acceptance rate between L&MICs countries and HICs and the fact that fewer studies are addressing parental vaccination hesitancy and the factors underlying the parents' decisions, and the urgency of research in L&MICs countries, we set our research questions as follows. “What proportion of the parents' population is willing to give the COVID-19 vaccine to their children in L&MICs?” And “what are the factors influencing their decision?”. Thereafter, this meta-analysis study aims at assessing the published literature on parents' acceptance in L&MICs in order to provide a more credible estimate of the proportion of parents accepting to vaccinate their children against COVID-19 as well as to identify determinants of COVID-19 vaccine parents' acceptance to vaccinate their children in L&MICs.

## Methods

### Design

This meta-analysis followed the PIO framework (Population, Intervention, and Outcome)used in the Evidence Based Medicine EBM (15) and the PRISMA 2020 statement on updated guidelines and the checklist for reporting systematic reviews (16). The target population (P) was the L&MICs population, of parents, caregivers, and guardians; intervention (I) was COVID-19 vaccination intention, and outcome (O) was COVID-19 vaccine hesitancy or acceptance among the target population.

### Inclusion and exclusion criteria

Studies included in this meta-analysis needed to confirm with the following criteria: published between December 2021 (first vaccine approval) till February 2022, use either quantitative or mixed methodology, express COVID-19 acceptance or hesitancy using proportions or absolute numbers, target L&MICs' population (parents, caregivers, and guardians) with accessibility to the COVID-19 vaccine, and finally, original peer-reviewed studies published in English.

On the other hand, exclusion criteria were: studies targeting populations other than parents, caregivers, or guardians; written in languages other than English; targeting HICs countries; using a qualitative approach.

### Search strategy

The search for the peer-reviewed studies was performed in three main databases: PubMed, Web of Science, and Cochrane Library. The following keywords–COVID-19, vaccine, hesitancy, and acceptance–were searched in the three databases using Boolean operators, truncation, and wildcard, where appropriate. The search term differed according to each database recommended search mechanism. Accordingly, the exact used search terms were:

#### PubMed

(“COVID-19”[MeSH Terms] OR “COVID-19 Vaccines”[MeSH Terms]) AND (“vaccine^*^”[Text Word] OR “Vaccines”[MeSH Terms]) AND (“vaccine acceptance”[Text Word] OR “Vaccination Hesitancy”[MeSH Terms]) AND (english[Filter]).

#### Cochrane

#1 MeSH descriptor: [Vaccination Hesitancy] explode all trees.

#2 (vaccin^*^ NEXT(hisitanc^*^ or acceptanc^*^)):ti,ab,kw (Word variations have been searched).

#3 #1 Or #2.

#4 MeSH descriptor: [COVID-19 Vaccines] explode all trees.

#5 (COVID^*^ NEXT (Vaccin^*^)):ti,ab,kw (Word variations have been searched).

#6 #4 OR #5.

#7 #3 AND #6.

#### Web of science

(((AB=(vaccin^*^ acceptance)) OR AB=(vaccin^*^ hesitancy)) AND AB=(COVID-19)) AND ((LA==(“ENGLISH”) NOT CU==(“USA” OR “ENGLAND” OR “ITALY” OR “CANADA” OR “FRANCE” OR “DENMARK” OR “KUWAIT” OR “PEOPLES R CHINA” OR “GERMANY” OR “AUSTRALIA” OR “SAUDI ARABIA” OR “CROATIA” OR “VENEZUELA” OR “U ARAB EMIRATES” OR “NEW ZEALAND” OR “ROMANIA” OR “CYPRUS” OR “HUNGARY” OR “LUXEMBOURG” OR “URUGUAY” OR “TRINIDAD TOBAGO” OR “SWITZERLAND” OR “SWEDEN” OR “SOUTH KOREA” OR “SINGAPORE” OR “QATAR” OR “PORTUGAL” OR “POLAND” OR “PANAMA” OR “OMAN” OR “NORWAY” OR “NETHERLANDS” OR “MALTA” OR “LITHUANIA” OR “JAPAN” OR “ISRAEL” OR “IRELAND” OR “GREECE” OR “FINLAND” OR “CZECH REPUBLIC” OR “CHILE” OR “BELGIUM” OR “BARBADOS” OR “BAHRAIN” OR “AUSTRIA”)) NOT (SE==(“LECTURE NOTES IN COMPUTER SCIENCE” OR “LECTURE NOTES IN OPERATIONS RESEARCH SPRINGER”) OR CF==(“16TH INTERNATIONAL CONFERENCE ON AVAILABILITY RELIABILITY AND SECURITY ARES” OR “EASTERN ALLERGY CONFERENCE”))).

The databases' search was done in the title and abstract.

### Data extraction and synthesis

Two authors (W.A.E.K.-M. and B.M.S.) independently identified and extracted the studies from the databases. The identified studies were imported into Zotero, a citation managing software that can locate duplicates and eliminate them. After removing the duplicates, the remaining studies were screened independently by both reviewers for eligibility. The screening was done in two steps. The first screening included title and abstract screening. Studies that passed the first screening went into the second step of screening where a full text article assessment was performed to confirm eligibility.

Studies which passed the two steps of screening were imported to an excel sheet table. The following information was extracted and entered to the excel sheet for each study: title, author, year of publication, sample size, proportions of the population that accepted/hesitated/refused the vaccine, and factors underlying parents' decisions.

### Critical appraisal

The chosen studies were evaluated by two independent reviewers. The studies were evaluated based on the five Cochrane criteria: bias resulting from deviations from intended interventions, bias resulting from missing outcome data, bias in measuring the outcome, bias resulting from the randomization process, and bias resulting from the selection of reported results. The studies met four of the criteria for validation. The randomization criterion was not used since the studies are not randomized control studies. The two reviewers discussed their findings and came to an agreement on the included ones.

### Meta-analysis

Data analysis was done in two steps. First, a descriptive analysis of the studies was performed including distribution of the studies among countries and among country classification, sample size, type of study, and data collected in each study. Second, a meta-analysis using R program. Meta-analysis was first done using the observed proportion method. This method assumes that parents' acceptance proportions follow a normal or binomial distribution (bell-shaped, centered around 0.5) with minimal variance which is seldom the case. Then second, using the Arcsine proportion model. The Arcsin proportion model is one of the statistical models used to transform proportions (i.e., the data used in this study) so that their distribution be more approximate to a normal distribution which is an assumption required by meta-analysis models (17). The Arcsin model acknowledges that proportional data derived from real studies are mostly not normally distributed (skewed) and that there is variance among different studies measures (e.g., proportion). Thus, accounting for skewness and stabilizing the variance among studies making it more constant. Both the observed proportions analysis and the Arcsine proportion analysis were done using: first, fixed effect model which assumes homogeneity of studies, and second, a random effect model. The random effect model is used due to the fact that the include studies are heterogeneous as proved in the fixed effect model. Finally, a brief analysis of the major factors underlying parents' COVID-19 vaccine acceptance was done.

## Results

The primary database search resulted in 806 studies. The number of studies was reduced to 742 after the first stage of screening and elimination of duplicates. Checking the title and abstract against the eligibility criteria, 712 studies were eliminated for one of the following reasons: being a qualitative study, not done in L&MICs, and the population used in the study is not parents. The remaining 30 studies passed through a full-text screening.

**Figure 1:**
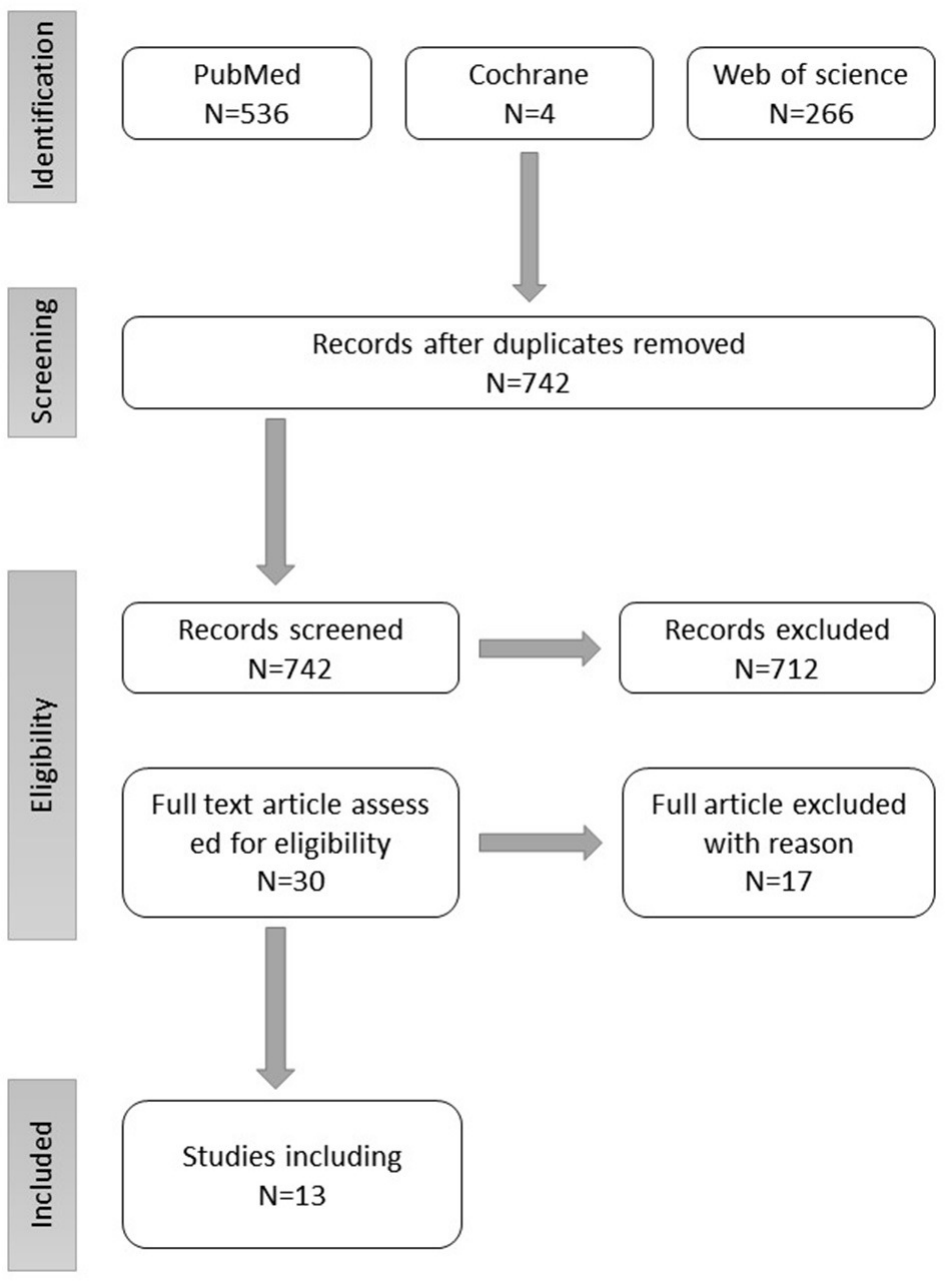
Search flow chart.

From the remaining 30 studies, seventeen studies were eliminated for combining both HICs and L&MICs in the same study calculations or for not collecting the same data as required by the methodology (Figure 1).

Thirteen studies were finally included in the meta-analysis. The studies included were cross-sectional type of studies. Only 10 out of 13 studies clearly stated the type of study in the text (Table 1). Two studies declared using a validated data collection tool (18, 19), while three provided the sources upon which their questionnaires were based (20, 21, 29). Certain variations were noticed among the studies: first, the study population. Seven out of thirteen studies did not have any specifications on the parents nor the children population in their sample, while two studies specified the age of participants as a criterion to choose participants (18, 23), one study specified that the participants were mothers (24), and two studies defined the parents' population to have children with specific medical conditions (25, 29). Second, the sample size, which ranged between 201 participants and 3079 participants among the studies. Third, the measurement term used within the study. Three main terms were used (acceptance, hesitancy, and refusal). Three out of thirteen studies used and provided data for all three terms (18, 25–27), while five studies used hesitancy only (20, 22, 23, 28, 29), and four used acceptances only (19, 21, 24, 30).

**Table 1:** Included articles' data and characteristics.

**References**	Type of study^*^	**Population**	**Country**	**Data collection tool**	**Sample size *N***	**Acceptance %**	**Hesitancy %**	**Refusal %**
Huynh et al. (20)	Cross sectional	General parents, children population	Vietnam	Questionnaire based on “health belief model”	1015		26.2	
Yılmaz et al. (26)	Cross sectional	General parents, children population	Turkey	Not based on a specific model	1135	36.3	35.6	28.1
Akgün et al. (25)	Cross sectional	Children with rheumatoid disease	Turkey	Not based on a specific model	201	41.8	45.8	12.4
Ali et al. (22)	Cross sectional	General parents, children population	Bangladesh	Not based on a specific model	2633		42.8	
Chinawa et al. (24)	Cross sectional	Mothers	Nigeria	Not based on a specific model	577	4.9		
Gönüllü et al. (30)	Cross sectional	Pediatricians	Turkey	Not based on a specific model	506	75		
Soysal et al. (18)	Not specified	Age 18–25 Not specified as parents	Turkey	Questionnaire based on “vaccine hesitancy questionnaire” by WHO	1033	68.8	11.4	3.1
İkiışık et al. (23)	Cross sectional	Age 20–85 Not specified as parents	Turkey	Not based on a specific model	384		89.6	
Bagateli et al. (21)	Not specified	General parents, children population	Brazil	Questionnaire based on “parents' attitude about childhood vaccine”	501	91		
Wang et al. (19)	Cross sectional	General parents, children population	China	Questionnaire based on “vaccine hesitancy questionnaire” by WHO	3079	52.4		
Zhang et al. (28)	Cross sectional	General parents, children population	China	Not based on a specific model	1788		52.5	
Ali et al. (29)	Cross sectional	Children with neurodevelopmental disorders	Bangladesh	Based on a questionnaire used in a published study	396		42.7	
Yigit et al. (27)	Not specified	General parents, children population	Turkey	Not based on a specific model	428	28.9	71.1	

As for the country where studies were performed, one can notice that 69% of the studies took place in Upper Middle-Income Countries UMICs (six in Turkey, two in China, and one in Brazil), while three studies were done in Lower Middle-Income Countries LMICs (two in Bangladesh and one in Vietnam) and one study in Nigeria, which is a Low-Income Country LIC (Table 1).

Looking at the required statistic (acceptance proportion) -which is either directly provided by the study or calculated through using other terms (e.g., hesitancy and refusal)- and the sample size, one can observe that it ranges between 4.9 and 91%, indicating huge variation among included studies (Table 1).

**Figure 2:**
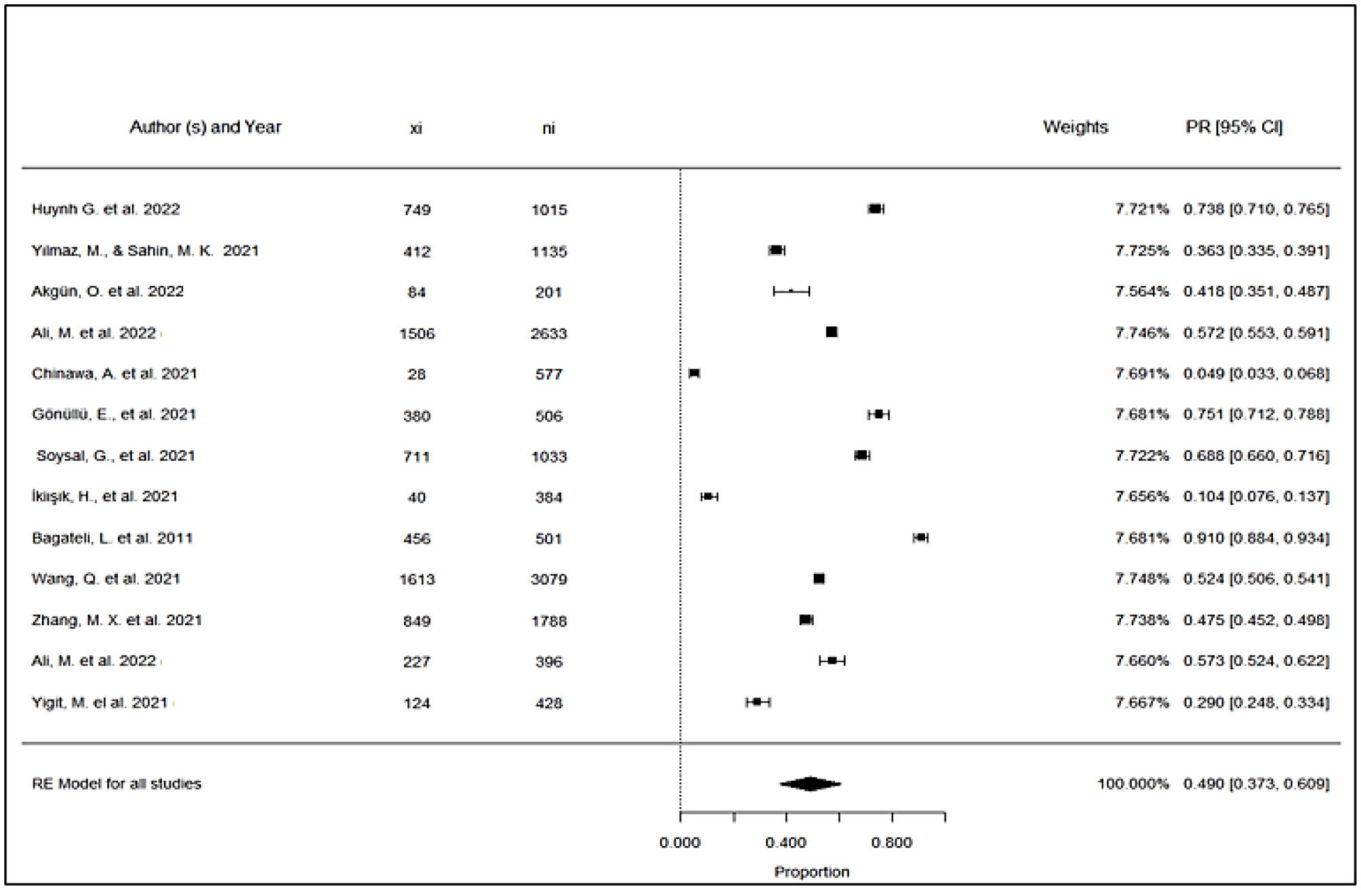
Forest plot: proportion of parents accepting to vaccinate their children against COVID-19 in L&MICs.

Although the 13 included studies have a wide range of sample sizes and resulted in different COVID-19 vaccine acceptance among parents, they are all significant. None of the studies crosses the vertical null effect line (Figure 2).

However, knowing that using the observed proportions method entails assuming that the proportions identified across the collection of studies follow a normal or binomial distribution with minimal variance, the Arcsine proportions method - which acknowledges that proportional data derived from real studies is mostly skewed and that there is variance among different studies measures- was used. The meta-analysis (Table 2) using the arcsine proportion models shows high heterogeneity among studies and the random effect model indicates the model is effective (*p* < 0.0001).

**Table 2:** Arcsine proportions of parents accepting to vaccinate their children against COVID-19 in L&MICs and effect sizes.

**References**	**Number of parents accepting the vaccine**	**Sample size**	**Weighted proportion**	**Variance**	**Standard error**	**Z statistics**	***P*-value**	**CI lower limit**	**CI upper limit**
Hyunh et al. (20)	749	1,015	1.0334	0.0002	0.0157	65.8444	< 0.0001	1.0026	1.0641
Yılmaz et al. (26)	412	1,135	0.6466	0.0002	0.0148	43.5689	< 0.0001	0.6175	0.6757
Akgün et al. (25)	84	201	0.7029	0.0012	0.0353	19.9317	< 0.0001	0.6338	0.7721
Ali et al. (22)	1,506	2,633	0.8576	0.0001	0.0097	88.0137	< 0.0001	0.8385	0.8767
Chinawa et al. (24)	28	577	0.2221	0.0004	0.0208	10.6705	< 0.0001	0.1813	0.2629
Gönüllü et al. (30)	380	506	1.0483	0.0005	0.0222	47.1636	< 0.0001	1.0048	1.0919
Soysal et al. (18)	711	1,033	0.9784	0.0002	0.0156	62.8951	< 0.0001	0.9480	1.0089
İkiışık et al. (23)	40	384	0.3286	0.0007	0.0255	12.8797	< 0.0001	0.2786	0.3786
Bagateli et al. (21)	456	501	1.2664	0.0005	0.0223	56.6925	< 0.0001	1.2226	1.3102
Wang et al. (19)	1,613	3,079	0.8093	0.0001	0.0090	89.8117	< 0.0001	0.7916	0.8269
Zhang et al. (28)	849	1,788	0.7602	0.0001	0.0118	64.2914	< 0.0001	0.7370	0.7834
Ali et al. (29)	227	396	0.8589	0.0006	0.0251	34.1836	< 0.0001	0.8096	0.9081
Yigit et al. (27)	124	428	0.5684	0.0006	0.0242	23.5169	< 0.0001	0.5210	0.6157
**Arcsin proportion model results**									
			**Estimate**		**Standard error**	**Z statistics**	***P*** **-value**	**CI lower limit**	**CI upper limit**
Fixed-effects model			0.8077		0.0042	188.9211	< 0.0001	0.7994	0.8161
Random-effects model			0.7758		0.0608	12.7515	< 0.0001	0.6566	0.8951
Test for heterogeneity: Q (degree of freedom = 12) = 2,281.3290									

When the results of arcsine proportions are transferred to normal proportions, the final effect size for these studies becomes 49.0% which happens to be significant with confidence limits of 37.3% and 60.9% within a confidence interval of 95% (Figure 2). Moreover, the studies have narrow confidence limits resulting in minimal differences in weights assigned to the different studies (Figure 2).

Concerning the factors affecting parents' decisions regarding the COVID-19 vaccination of their children, they are numerous and vary among studies (Annex 1). The most common factor for parents' hesitancy to vaccinate their children against COVID-19 is their concerns about vaccine efficacy, safety, and possible side effects. This factor was mentioned in 11 studies out of 13. On the other hand, the most common factor for parents' acceptance to vaccinate their children is their conviction that the vaccine is needed to control COVID-19 and end the pandemic. Lastly, parents refused to vaccinate their children due to distrust of vaccine manufacturing companies and vaccine safety and efficacy.

## Discussion

Given the COVID-19 gravity, assessing vaccine hesitancy has become very crucial for governments and policymakers. Persuading people to be vaccinated is essential to protect themselves and others by limiting the global spread. An important category regarding vaccine hesitancy is the parents or caregivers who influence the vaccination process of their children. The number of studies included in this meta-analysis, and their distribution indicates the weak attention given to parents' behavior against COVID-19 vaccine in L&MICs especially in LICs (24). Taking into account the higher fertility rate and higher proportion of children in L&MICs, concentrating on parents' behavior might be a key factor in fighting against the COVID-19 pandemic.

It is worth noting that the most common announced factor for parents' acceptance to vaccinate their children is that the vaccine is needed to control COVID-19 and end the pandemic which indicates a certain level of these parents' awareness regarding COVID-19 and the vaccine (23, 25, 26, 30). Other acceptance factors mentioned in the studies are equally important (Annex 1). Parents accepting to vaccinate their children are found to be the parents who accept the notion of vaccines in general, they believe that the benefit of vaccination outweighs its harm. These are parents who are vaccinated yearly against influenza and who follow vaccination regimes for their children (18, 30). This signifies that parents' acceptance to vaccinate their children against COVID-19 is more related to the fact that they believe in the benefit of vaccine rather than the fear of COVID-19 itself.

As for factors related to COVID-19 vaccine hesitancy and refusal, the most commonly identified factor is the uncertainty about COVID-19 vaccine efficacy, safety and possible side effects (18, 22, 26, 27, 29). This factor highlights the lack of trust that the parents have in their governments as well as vaccine manufacturers (27, 29). This factor can be related to the final proportion of parents accepting to vaccinate their children against COVID-19, which is around 49% (Figure 2).

The above proportion is lower than the worldwide estimated proportion. According to a similar meta-analysis done on a global level (i.e., HICS, MICs and LICs are included), parents' willingness to vaccinate their children ranges between 25.6 and 92.2% worldwide; and the overall proportion of parents intending to vaccinate their children against COVID-19 is 60.1% (31). This may be related to various reasons. In African countries, the demand for vaccine decreased due to public concerns about the possibility of COVID-19 exposure when receiving vaccination. This concern has equally affected parental health-seeking behavior resulting in lower parental acceptance (32). Other reasons for the lower parental acceptance in L&MICs might be related to factors such as trust in authorities and subsequently trust in the type of vaccine provided by the country. Also, some L&MICs might concentrate less on health promotion strategies and the availability of data on COVID-19 vaccines' safety and efficacy compared to HICs. Finally, the economic status and the educational background may play a role in the reduced parental acceptance. People in some of these countries are extremely poor with low education hence might be ignorant of the benefits of the vaccine. Others might trust traditional medical practice over conventional medicine resulting in lower acceptance rate.

Given that educational background, economic status, and available health promotion strategies all influence individual perceptions of vaccines, addressing the fears of anti-vaxxers is of great importance, especially in the presence of inconsistent information regarding vaccine safety and efficacy that may be present on different live or online networks. Healthcare providers can help parents overcome their fears about vaccinating themselves and their children. Healthcare providers need to have the proper knowledge and the essential skills to address these fears correctly.

The fact that there are different terms used to address parents' behavior concerning vaccinating their children against COVID-19 indicates that researchers ought to consider adding a clear definition of the terms they use in their studies to avoid confusions, especially that the terms acceptance and refusal are present in the definition of vaccine hesitancy. Moreover, not all studies use a validated data collection tool, resulting in variation in the data collected, especially the factors underlying parents' decisions, which in turn renders comparing between the studies and stating unified factors for the L&MICs parents' decisions more difficult.

This study has its limitations such as language bias. Only English studies were eligible to be included, which means that many non-English studies are missing. Also, the timeframe for the study. Although it is necessary to set a timeframe for the study as the pandemic is still ongoing and more studies will come out, setting a timeframe here limits the number of studies included. These limitations call for further research that can include other languages published studies over a longer period of time.

## Conclusion

This meta-analysis concludes that the proportion of parents in L&MICs accepting to vaccinate their children against COVID-19 is 49%, and the major reason for their acceptance is that they believe that COVID-19 vaccine is fundamental to the fight against the pandemic. To increase parental acceptance, responsible authorities should concentrate on increasing their population trust in the government as well as in the vaccine manufacturers. In addition, authorities ought to concentrate on increasing acceptance of the vaccine idea in general through highlighting the need for the vaccine to end the pandemic and assuring the efficacy and safety of the vaccine. Further research on parental behavior concerning vaccinating of their children is needed in L&MICs especially in LICs.

## Data availability statement

The original contributions presented in the study are included in the article/Supplementary material, further inquiries can be directed to the corresponding author.

## Author contributions

WA participating in conceptualization, methodology framing, validation, formal analysis, investigation, writing the original draft, reviewing, and editing of the manuscript. BS participated in methodology framing, validation, investigation, writing, reviewing, and editing of the manuscript. HE-F participated in validation, reviewing, and editing of the manuscript. SC participating in conceptualization, methodology framing, validation, formal analysis, investigation, reviewing, and editing of the manuscript. All authors have read and agreed to the published version of the manuscript.
