# Supplementary material for: COVID-19 vaccine hesitancy among parents in Low- and Middle-Income Countries: A meta-analysis
Source: Front Public Health. 2023 Feb 15;11:1078009. doi: 10.3389/fpubh.2023.1078009 (PMC10010145; doi:10.3389/fpubh.2023.1078009)
Supplement: Supplementary file 1 [file Table_1.docx]

Annex 1

| Article | Factors |
| --- | --- |
| Huynh G. et al. 2022 [18] | Reasons for parent’ hesitancy   1. concerns about side effects 2. vaccine safety |
| Yılmaz, M., & Sahin, M. K. 2021[24] | factors for parents’ willingness to allow vaccination:   - need for COVID-19 control - benefits of the COVID-19 vaccine outweighing its potential harm - to protect not only their own families but also others   factors for reluctance to allow vaccination:   - lack of sufficient scientific studies - concern about safety and side effects - potential inefficacy of the vaccine due to mutations |
| Akgün, O. et al. 2022 [23] | factors affecting parents' acceptance of vaccines for their children were as follows:   - “Receiving anti-rheumatic medications regularly - previous history of getting special recommended vaccines - relying on vaccines for ending pandemic complying with the pandemic measures entirely |
| Ali, M. et al. 2022 [27] | Hesitancy factors:   - parents of 0–4-year-old children’s parents of girls - young parents - Muslims parents who received college education - unemployed parents - parents with a household income of <৳15 000 those who lived in their own house - came from a village lived in the north zone - tobacco users’ parents politically affiliated with opposition parties - participants who did/will not vaccinate their child with regular vaccines (other than COVID-19) available under government programs - those who did not believe in the effectiveness of the COVID-19 vaccine for Bangladeshi children - those who did not/will not receive the COVID-19 vaccine for themselves Parents who were not likely to believe that their children or a family member could be infected with COVID-19 in the next year those not concerned about their children or a family member getting COVID-19 in the next year |
| Chinawa, A. et al. 2021 [22] | - believed they could be infected with the COVID-19 or are aware of someone that died from COVID-19 |
| Gönüllü, E., et al. 2021 [28] | Acceptance factors:   - believe that effective vaccine will be developed - accepted to be enrolled as a subject in phase 2 clinical COVID-19 vaccine trial - thought that COVID-19 vaccine should be mandatory - thought that COVID-19 vaccine passport should be used in entrance to school and travel - were vaccinated with influenza vaccine in year 2019 - who wanted to get influenza vaccine shot in year 2020 |
| Soysal, G., et al. 2021 [16] | Hesitancy factors   - age - advanced and negative information was received on childhood vaccines   Acceptance factors   - thought that childhood vaccines could protect against severe diseases than those who had no idea about this subject |
| İkiışık, H., et al. 2021[21] | Factors affecting vaccine acceptance   - the perception of risk - age |
| Bagateli, L. et al. 2011 [19] | Hesitancy factors   - the caregivers were concerned about serious side effects of the vaccines - had some concerns about their safety |
| Wang, Q. et al. 2021 [17] | - willing: to “protecting the people around” - unwillingness: “concern about side effects” |
| Zhang, M. X. et al. 2021 [26] | Hesitancy factors:   - parents with children under 18 years of age - lower knowledge scores about COVID-19 vaccination - lower awareness of the permission of vaccinating children - hesitancy to inoculate themselves |
| Ali, M. et al. 2022[20] | Hesitancy factors:   - parents who lived in the northern zone - those who thought vaccines would not be safe and effective for Bangladeshi children - those who were either not vaccinated or did not receive the COVID-19 vaccine themselves - those who said that they or their family members had not tested positive for COVID-19 - those who did not lose a family member to COVID-19 - parents who were not likely to believe that their children or a family member could be infected with COVID-19 the following - who were not concerned at all about their children, or a family member being infected the following year |
| Yigit, M. el al. 2021 [25] | Refusal factors:   - avoiding possible vaccine side effects - not knowing the precise effectiveness of the vaccine - distrust in vaccines from abroad - concerns about excipients in the vaccine - not believing in the effectiveness of vaccines - not being afraid or anxiety about COVID-19 infection - distrust in domestic vaccines - thinking he will not have COVID-19 again - religious reasons - believing the virus will mutate so that the vaccine will be ineffective - distrust in companies developing vaccines - thinking that the vaccines might contain microchips |
